# The Prevalence of Sexually Transmitted Infections among Male Patients at a Dermato-Venereology Outpatient Clinic in Gdańsk, Poland: Findings from a Single-Center Study

**DOI:** 10.3390/jcm13133736

**Published:** 2024-06-26

**Authors:** Damian Kadylak, Justyna Czarny, Roman Janusz Nowicki, Małgorzata Sokołowska-Wojdyło

**Affiliations:** 1 Department of Dermatology, Venereology and Allergology, Faculty of Medicine, Medical University of Gdańsk, Smoluchowskiego 17 Street, 80-214 Gdańsk, Poland; 2 Department of Dermatology, Venereology and Allergology, University Clinical Centre, Smoluchowskiego 17 Street, 80-214 Gdańsk, Poland

**Keywords:** *Neisseria gonorrhoeae*, *Chlamydia trachomatis*, *Mycoplasma genitalium*, *Trichomonas vaginalis*

## Abstract

**Background**: Sexually transmitted infections (STIs) are a significant public health concern worldwide, yet data on their prevalence and epidemiology, particularly in Central and Eastern Europe, remain scarce. This study aimed to assess the prevalence, anatomical localization, symptomatic/asymptomatic course, and co-infection patterns of STIs among men. **Methods**: This retrospective study analyzed data collected between May 2021 and July 2023, including sociodemographic, sexual behavior, and clinical data from 139 male participants. Molecular polymerase chain reaction (PCR) tests were conducted for *Neisseria gonorrhoeae* (NG), *Chlamydia trachomatis* (CT), *Mycoplasma genitalium*, and *Trichomonas vaginalis*. **Results**: Of the participants, 36% tested positive for at least one STI, with the urethra being the most common site of infection. NG and CT were the most prevalent infections. The majority of infections were asymptomatic, highlighting the importance of comprehensive screening, especially in high-risk populations like men who have sex with men (MSM). **Conclusions**: This study emphasizes the need for targeted screening strategies, particularly for extragenital STIs, and underscores the role of MSM in STI epidemiology. The findings highlight the importance of routine screening, even for asymptomatic individuals, to effectively control STI spread. Future research should validate and expand upon these findings to enhance STI prevention and management efforts.

## 1. Introduction

Sexually transmitted infections (STIs) pose a significant global public health concern, impacting millions of individuals daily [1]. Early detection and treatment of STIs are crucial, not only for the individual health of the patient but also to prevent the transmission to sexual partners.

Globally, *Chlamydia trachomatis* (CT), *Neisseria gonorrhoeae* (NG), and *Trichomonas vaginalis* (TV) are the most prevalent and treatable bacterial STIs, accounting for an estimated 367 million new cases worldwide in 2020 [2]. In the European Union/European Economic Area alone, 216,508 confirmed cases of CT infection were reported in 2022, with a crude notification rate of 88 cases per 100,000 in the population [3]. In the same year, 70,881 confirmed cases of NG were reported, with a crude notification rate of 17.9 cases per 100,000 in the population [4]. STI prevalence studies in the Central and Eastern European regions are limited and are especially scarce in the non-European Union regions of Europe, with a notable lack of epidemiological data or a need for more extensive studies on the incidence of CT, NG, *Mycoplasma genitalium* (MG), and TV in asymptomatic individuals [5].

The burden of STIs in Poland is unknown for several reasons. Firstly, many STIs often manifest as asymptomatic. Secondly, achieving an accurate diagnosis is challenging due to limited accessibility to adequate testing equipment, and infections are frequently treated empirically without proper diagnostics. Lastly, many cases of infection are not reported. Universal mandatory reporting for epidemiological surveillance indicates a significant increase in the incidence of STIs in Poland over recent years. Comparing 2022 and 2023 data from the Centre for Health Information Systems within the Ministry of Health, there was an 118% increase in new cases of NG (from 630 cases in 2022 to 1372 in 2023) and an 89% increase for CT (from 517 cases in 2022 to 977 in 2023) [6,7]. Data on the incidence of MG and TV infections in Poland are unknown because they are not subject to mandatory reporting.

## 2. Aim

This study aimed to assess the prevalence of STIs and determine their anatomical localization, symptomatic and asymptomatic course, and co-infection patterns among men attending the Dermato-Venereology Outpatient Clinic in Gdańsk (Gdańsk, Poland). This retrospective study was conducted between May 2021 and July 2023. We used sociodemographic characteristics, sexual behavior data, and clinical data routinely collected at the Dermato-Venereology Outpatient Clinic in Gdańsk.

## 3. Materials and Methods

### 3.1. Inclusion Criteria

Males aged 16 years and older, who sought care at our STI clinic due to suspected or confirmed STIs or engagement in high-risk sexual behaviors, were included in this study. High-risk behaviors were defined as engaging in unprotected intercourse, having multiple sexual partners, participating in paid sexual encounters, or engaging in chemsex.

### 3.2. Population and Data Collection

**Figure 1:**
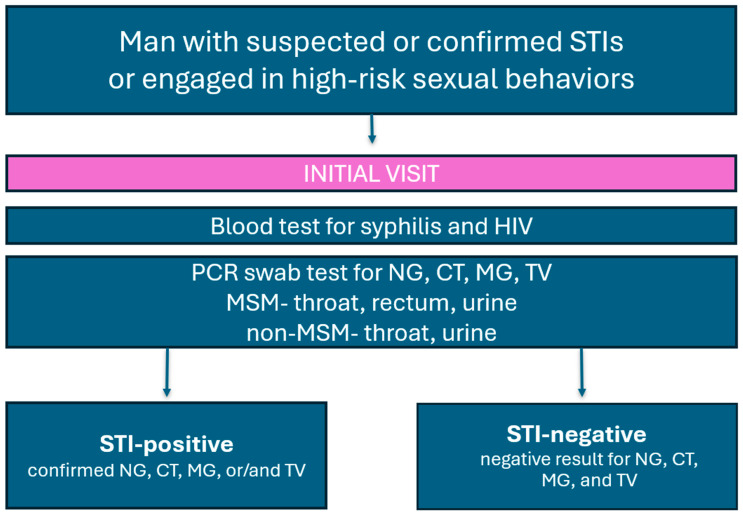
Study design.

This study involved 139 male participants. Data were extracted from existing clinical records and patient interviews to include information about the participant’s sexual behaviors, potential risk factors, and types of samples provided. Molecular polymerase chain reaction (PCR) tests were conducted during the initial visit for all patients who consented to a screening (Figure 1). Based on documented sexual encounters, men who have sex with men (MSM) provided rectal and throat swabs, as well as urine samples, and non-MSM participants provided throat swabs and urine samples. All study participants underwent peripheral blood sampling to test for syphilis and HIV. The STI-positive group was defined as the group in which at least one infection with NG, CT, MG, or TV was detected by molecular testing. The STI-negative group was defined as the group in which no infection with NG, CT, MG, or TV was detected by molecular testing.

### 3.3. Sample Analysis

All specimens were screened for CT, NG, MG, and TV by PCR examinations using the Fast Track Diagnostics multiplex Real-Time PCR (Fast Track Diagnostics Ltd., now part of Siemens Healthineers, Silema, Malta) according to the manufacturer’s instructions. The samples were not pooled.

### 3.4. Statistical Analysis

The calculations were performed using IBM SPSS Statistics 23 software. Pearson’s χ^2^ test was employed, which is a non-parametric test used to investigate the relationship between two variables measured on a qualitative scale. A statistically significant result (*p* < 0.05) indicated the presence of a relationship between the variables.

## 4. Results

### 4.1. Participant Characteristics

Between May 2021 and July 2023, 139 participants took part in the study, including 106 MSM and 31 non-MSM individuals (Table 1). The participants’ ages ranged from 16 to 54 years, with an overall median age of 30. The average age was 31 years, with a standard deviation of 8.6 years. MSM constituted 76.26% (*n* = 106) of the individuals in our cohort (74.7% and 82% in the STI-negative and STI-positive groups, respectively). Regarding HIV status, 18% (*n* = 16) of participants in the STI-negative group and 16% (*n* = 8) in the STI-positive group were HIV-positive. Current pre-exposure prophylaxis (PrEP) usage was reported by 6.7% (*n* = 6) and 14% (*n* = 7) of participants in the STI-negative and STI-positive groups, respectively. Overall, 85 participants (61%) in the study groups had a co-infection with syphilis. Of those 85, syphilis co-infection was diagnosed in 26 patients (52%) in the STI-positive group. No statistically significant disparity was detected between the STI-positive and STI-negative groups regarding the overall age distribution, number of MSM, HIV infection, PrEP usage, or concurrent syphilis infection.

### 4.2. STIs in Anatomic Sites

**Figure 2:**
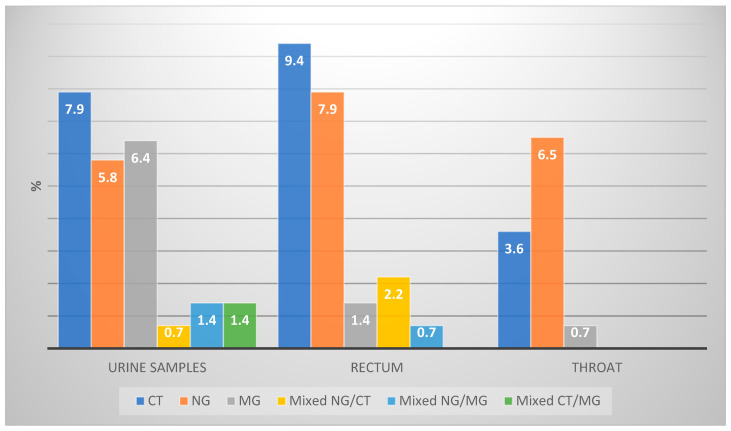
Representation of STIs by anatomical site in the study group.

In the study cohort, 36% (*n* = 50) of patients tested positive for at least one STI pathogen at a single anatomical site, with 7.9% of infections occurring at multiple sites. The most prevalent sites of infection were the urethra (16.5%), followed by the rectum (15.8%) and the throat (10.8%) (Table 2). Urethral infections were most commonly caused by CT (34.8%), followed by both NG and MG (21.7% each), then mixed infections with NG/MG and CT/MG (8.7% each), and finally with NG/CT (4.3%) (Figure 2). In the rectum, CT was the most prevalent (45.5%), followed by NG (31.8%), mixed infections with NG/CT (13.6%), and then both MG and mixed NG/MG infections (4.5% each). In the throat, NG was the most common (60%), followed by CT (33.3%) and MG (6.7%).

The distribution of pathogen infections in relation to sexual behaviors displayed some noteworthy disparities. At least one of the STIs was present in at least one location in 38.7% of MSM and in 29% of non-MSM individuals. Multi-site infections were detected in 9.4% of MSM (NG and CT) and in 3.2% of non-MSM individuals (NG). In the non-MSM group, extragenital STI infections were detected in only 3.2% of cases, whereas in the MSM group, this figure rose to 34.9%. In MSM, NG, CT, and MG were prevalent in 18.9%, 16%, and 10.4% of individuals, respectively, while in the non-MSM group, the prevalence rates were 9.7%, 19.4%, and 3.2%, respectively. The predominant pathogen among non-MSM individuals was CT, with infections primarily occurring in the urethra and being characterized by classic symptoms on presentation in 83.33% of cases. In contrast, the most prevalent pathogen detected in the MSM group was NG, with infections predominantly found in the rectum and characterized by having classic overall symptoms at presentation in 36.36% of cases.

### 4.3. Symptomatic and Asymptomatic Course

**Figure 3:**
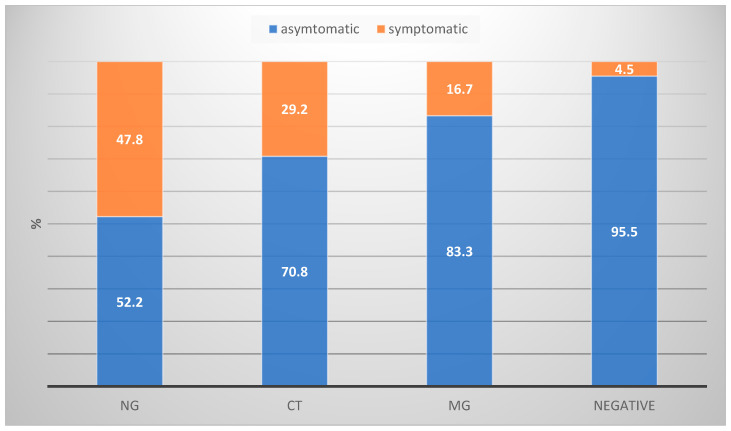
Representation of symptomatic and symptomatic STIs in the study group.

In our study group, we observed varying courses of infection—symptomatic and asymptomatic—depending on the location and type of STI. NG infections were symptomatic in 47.8% of individuals (Figure 3, Table 3). Symptomatic NG infections were more prevalent in both the genital and extragenital regions, with rates of 87.5% and 38.9%, respectively, and both findings were statistically significant. For CT infections, 29.2% of individuals were symptomatic. In the genital regions, 54.5% of CT infections were symptomatic, while only 6.7% were symptomatic in extragenital areas. A significant association was found between symptomatic CT infections and the genital area, but not for extragenital infections. Regarding MG infections, only 22.2% of genital infections were symptomatic, while all extragenital infections were asymptomatic. Our study found no significant correlation between the presence of MG and a symptomatic presentation. Furthermore, if screening had been limited to symptomatic urogenital infections, our study revealed substantial omissions: 69.57% for NG infections, 73.91% for CT infections, and 83.33% for MG infections.

### 4.4. Neisseria Gonorrhoeae

NG infections were identified in 16.5% of the cohort. The rectum was the primary NG infection site in 7.9% of participants, followed by the throat in 6.5% and the urethra in 5.8%. Notably, 3.6% of participants had NG infections in multiple sites: urethra/throat (1.4%), rectum/throat (1.4%), and urethra/rectum (0.7%). Stratifying by sexual behavior, NG was detected in 18.9% of MSM and 9.7% of non-MSM participants. Among MSM, the distribution of NG infections included 10.4% of infections occurring in the rectum, 7.5% in the throat, and 4.7% in the urethra. For non-MSM participants, the urethra was the primary NG infection site in 9.7% of cases, followed by the throat in 3.2% of cases.

### 4.5. Chlamydia Trachomatis

CT was detected in 16.5% of participants. It was most prevalent in the rectum (9.4%) and the urethra (7.9%), with a lower prevalence found in the throat (3.6%). Among participants, 4.3% had CT infections at multiple sites: rectum/throat (2.2%), urethra/rectum (1.4%), and urethra/throat (0.7%). Stratifying by sexual behavior, CT was found in 16% of MSM and 19.4% of non-MSM participants. In MSM, CT was most prevalent in the rectum (12.3%), followed by the throat and urethra (both 4.7%). In non-MSM participants, CT was solely detected in the urethra (19.4%).

### 4.6. Mycoplasma Genitalium

MG was detected in 8.6% of participants. The urethra was the primary MG infection site with 6.5% of participants affected, followed by the rectum with 1.4% and the throat with 0.7%. Notably, there were no cases of MG infection across multiple locations. MG was found in 10.4% of MSM and 3.2% of non-MSM participants. Among MSM, MG was identified in the urethra in 7.5% of this group’s participants, followed by 1.9% in the rectum and 0.9% in the throat. In non-MSM participants, MG was exclusively identified in the urethra (3.2%).

### 4.7. Trichomonas Vaginalis

No TV infections were detected in our study population.

## 5. Discussion

Recent studies highlight the importance of molecular screening in extragenital locations, particularly in asymptomatic patients [8,9]. While genital infections often present with symptoms, extragenital infections are frequently asymptomatic, making screening in these sites particularly significant [9]. Jansen et al. observed that molecular testing limited to individuals with symptomatic urethritis may detect only one-third of all diagnosed infections [8]. Similarly, Ota et al., in a study involving 176 individuals with suspected extragenital NG and/or CT infections, found that testing only urethral or urine samples missed 60% of pharyngeal NG infections and 84% of pharyngeal CT infections [10]. In our study, we similarly observed that limiting screening to symptomatic urethritis resulted in the overall omission of 69.57% of NG infections, 73.91% of CT infections, and 83.33% of MG infections. Given the substantial proportion of extragenital and asymptomatic infections, our findings strongly support the widespread implementation of STI screening, especially for MSM, emphasizing screening at all three anatomical sites. Moreover, untreated infections with NG and CT have been demonstrated in some studies to elevate the genital viral load of HIV, potentially facilitating transmission [11].

The emergence and spread of antimicrobial resistance (AMR) in NG is a serious threat to the treatment and control of gonorrhoea [12,13]. Extragenital infections, anorectal and especially pharyngeal, may also play an important role in the development of resistant strains, as NG interacts and exchanges genetic material with other co-infections in these anatomical sites [14]. The presence of other commensal Neisseria species in the oropharynx can act as a reservoir of resistance against NG [15,16,17,18,19,20]. Lewis et al. further emphasized the importance of addressing oropharyngeal gonorrhea to delay the emergence of drug-resistant strains [15]. On the other hand, Teker et al. demonstrated that 14.5% of patients with a rectum NG infection, 18.7% of patients with a pharyngeal NG infection, 23.1% of patients with a vaginal NG infection, and 32.1% of patients with a urethral NG infection spontaneously cleared their asymptomatic infections [21]. This raises questions about the appropriateness of screening studies for STIs and substantiates the argument that not all individuals require treatment. In light of the escalating AMR associated with NG, a prudent approach would be a reduction in the indiscriminate use of antibiotics.

Most of the available comparative data in the literature pertains to the MSM population. Streeck et al., in their study in Germany, reported an overall STI prevalence of 35.5% among MSM, with MG being the most common pathogen (19.0%), followed by CT (12.8%) and NG (10.1%) [22]. Our study demonstrated a slightly higher overall STI prevalence of 38.7% among MSM, with NG, CT, and MG being prevalent at 18.9%, 16%, and 10.4%, respectively. Regarding anatomical site-specific infections, Streeck et al. reported the highest STI prevalence at the anorectal site (25.9%), followed by the pharyngeal (10.3%) and urethral (9.0%) sites. MG was the most common anorectal infection (13.4%), followed by CT (10.2%) and NG (6.5%). In our study, among MSM, CT was the most prevalent anorectal infection (12.3%), followed by NG (10.4%) and MG (1.9%). Pharyngeal infections in Streeck’s study were dominated by NG (5.8%), MG (3.3%), and CT (2.4%), whereas our study showed NG as the most prevalent (7.5%), followed by CT (4.7%) and MG (0.7%). For urogenital infections, Streeck et al. reported MG (6%) as the most frequent pathogen, with lower rates for CT (2.6%) and NG (1%). Our study found MG in 7.5% of participants, with prevalence rates of 4.7% for both CT and NG. Both Streeck et al. and our study highlighted the importance of detecting multi-site infections. Streeck et al. reported that 1.7% of participants had STIs at all three anatomical sites, with 6.1% testing positive for STIs at both the anorectal and pharyngeal sites. Our study found multi-site infections in 9.4% of MSM, emphasizing the need for comprehensive multi-site screening to capture the full extent of the STI burden.

Minetti et al. found NG to be the most prevalent infection (16.6%) among MSM in Lisbon, followed by CT (13.2%) and MG (10.3%) [23]. Our findings for NG (18.9%) and CT (16%) were somewhat higher, while the prevalence of MG (10.4%) was consistent with Minetti’s data. In another study conducted by Szetela et al. in Poland, involving a cohort of 103 randomly selected MSM considered to be at a heightened risk of HIV infection, oropharyngeal NG infections were found to be the most prevalent (10.78%), while our study identified rectal NG infections as the most common (10.4%) [24]. For CT infections, Szetela et al. reported a more even distribution across all sites (oropharyngeal 9.9%, urethral 8.82%, rectal 10.89%), whereas our study found a significantly higher prevalence in the rectum (12.3%) compared to the throat and urethra (both 4.7%).

In 2016, the World Health Organization estimated a global prevalence of 0.6% for TV in men. TV is seldomly recognized as the cause of urethral and/or rectal infections in MSM. In the study conducted by Minetti et al., only 0.2% of MSM were found to be infected with TV [23]. Studies, such as one involving 678 MSM and another longitudinal study with 600 MSM, reported no cases of TV infection [25]. The lower prevalence observed in MSM may be attributed to the pathogen’s higher levels of persistence in the female urogenital tract [8]. Our study, encompassing both MSM and non-MSM groups, found no instances of TV infection. Based on these study results, it is not advisable to include TV in a routine STI testing scheme for MSM.

However, our study has several limitations. Firstly, the study is retrospective and single-center, which may result in the sample not being representative of all men in Poland due to our specific selection criteria. Secondly, a significant proportion of participants had concurrent syphilis diagnoses, which challenges the generalizability of our findings. Thirdly, the relatively small sample size, especially in the non-MSM group, may not capture the full spectrum of STI prevalence and behaviors. Finally, in the non-MSM group, rectal swabs were not conducted, limiting our understanding of potential infections in that anatomical site, including transmission from other locations.

## 6. Conclusions

The prevalence of STIs remains a significant concern, particularly due to their often asymptomatic nature. This emphasizes the critical role of comprehensive screening programs, especially for extragenital STIs, which are particularly prevalent among MSM. Our study underscores the importance of implementing targeted screening strategies tailored to specific anatomical sites, particularly in high-risk populations like MSM. One of the most significant advantages of obtaining swabs from three anatomical sites is the improved detection rate of STIs that might otherwise remain undiagnosed and untreated. Asymptomatic infections have the potential to be transmitted to sexual partners, thereby perpetuating the cycle of infection. Our findings also shed light on the non-MSM group, emphasizing that they too play a significant role in STI epidemiology. The limited research on this demographic underscores the importance of our findings and the need for more comprehensive studies in the future. The observation that many patients visited our clinic with symptoms or with other sexually transmitted diseases, and that screening was conducted as an additional measure, raises questions about the potential number of individuals harboring asymptomatic infections who are not seeking medical attention. This highlights the need for broader community education on the importance of routine screening, particularly for asymptomatic individuals, to effectively control and manage the spread of sexually transmitted infections. Moving forward, future research endeavors should prioritize validating and expanding upon our findings using larger and more diverse cohorts, thereby enriching our understanding of and approach to STI prevention and management.

## Figures and Tables

**Table 1:** Characteristics of the study groups.

Parameter	STI				*V*	*p*
	Negative (*n* = 89)		Positive (*n* = 50)			
HIV infection					0.03	0.767
No	73	82.0%	42	84.0%		
Yes	16	18.0%	8	16.0%		
PrEP users					0.12	0.158
No	83	93.3%	43	86.0%		
Yes	6	6.7%	7	14.0%		
Previous STI					0.10	0.248
No	62	69.7%	30	60.0%		
Yes	27	30.3%	20	40.0%		
Sexual behaviors					0.08	0.326
non-MSM	22	25.3%	9	18.0%		
MSM	65	74.7%	41	82.0%		
miising values: 2						
Syphilis coexisting					0.14	0.097
No	30	33.7%	24	48.0%		
Yes	59	66.3%	26	52.0%		
Symptoms					0.40	0.000
No	85	95.5%	33	66.0%		
Yes	4	4.5%	17	34.0%		

**Table 2:** Distribution of STIs and occurrence in individual anatomical locations.

		NG			CT			MG			Any-STI			Multiple Pathogens		
		All *n* = 139	MSM *n* = 106	Non-MSM *n* = 31	All *n* = 139	MSM *n* = 106	Non-MSM *n* = 31	All *n* = 139	MSM *n* = 106	Non-MSM *n* = 31	All *n* = 139	MSM *n* = 106	Non-MSM *n* = 31	All *n* = 139	MSM *n* = 106	Non-MSM *n* = 31
Urine	*n*	8	5	3	11	5	6	9	8	1	23	14	9	5	4	1
	% (95%Cl)	5.8% (1.9–9.6)	4.7% (0.7–8.8)	9.7% (0–20.1)	7.9% (3.4–12.4)	4.7% (0.7–8.8)	19.4% (5.4–33.3)	6.5% (2.4–10.6)	7.5% (2.5–12.6)	3.2% (0–9.4)	16.5% (10.4–22.7)	13.2% (6.8–19.7)	29% (13.1–45)	3.6% (0.5–6.7)	3.8% (0.1–7.4)	3.2% (0–9.4)
Throat	*n*	9	8	1	5	8	0	1	1	0	15	14	1	0	0	0
	% (95%Cl)	6.5% (2.4–10.6)	7.5% (2.5–12.6)	3.2% (0–9.4)	3.6% (0.5–6.7)	7.5% (2.5–12.6)		0.7% (0–2.1)	0.7% (0–2.1)		10.8% (5.6–15.9)	13.2% (6.8–19.7)	3.2% (0–9.4)			
Rectum	*n*	11	11	0	13	13	0	2	2	0	22	23	0	4	4	0
	% (95%Cl)	7.9% (3.4–12.4)	10.4% (4.6–16.2)		9.4% (4.5–14.2)	12.3% (6–18.5)		1.4% (0–3.4)	1.9% (0–4.5)		15.8% (9.8–21.9)	21.7% (13.9–29.5)		2.9% (0.1–5.7)	3.8% (0.1–7.4)	
Any site	*n*	23	20	3	23	17	6	12	11	1	50	41	9	8	7	1
	% (95%Cl)	16.5% (10.4–22.7)	18.9% (11.4–26.3)	9.7% (0–20.1)	16.5% (10.4–22.7)	16% (9.1–23)	19.4% (5.4–33.3)	8.6% (4–13.3)	10.4% (4.6–16.2)	3.2% (0–9.4)	36% (28–43.9)	38.7% (29.4–48)	29% (13.1–45)	5.8% (1.9–9.6)	6.6% (1.9–11.3)	3.2% (0–9.4)
Multiple sites	*n*	5	4	1	6	6	0	0	0	0	11	10	1	1	1	0
	% (95%Cl)	3.6% (0.5–6.7)	3.8% (0.1–7.4)	3.2% (0–9.4)	4.3% (0.9–7.7)	5.7% (1.3–10.1)					7.9% (3.4–12.4)	9.4% (3.9–15)	3.2% (0–9.4)	0.9% (0–2.8)	0.9% (0–2.8)	

**Table 3:** The occurrence of disease symptoms in patients with STIs based on anatomical location.

Parameter		*N*	Symptoms				*V*	*p*
			No (*n* = 118)		Yes (*n* = 21)			
**Neisseria gonorrhoeae**	Genital and extragenital localisation						0.41	0.000
	Negativ	116	106 (91.4%)		10 (8.6%)			
	Positiv	23	12 (52.2%)		11 (47.8%)			
	Genital localisation						0.50	0.000
	Negativ	131	117 (89.3%)		14 (10.7%)			
	Positiv	8	1 (12.5%)		7 (87.5%)			
	Extragenital localisation						0.26	0.003
	Negativ	121	107 (88.4%)		14 (11.6%)			
	Positiv	18	11 (61.1%)		7 (38.9%)			
**Chlamydia trachomatis**	Genital and extragenital localisation						0.18	0.034
	Negativ	115	101 (87.8%)		14 (12.2%)			
	Positiv	24	17 (70.8%)		7 (29.2%)			
	Genital localisation						0.32	0.000
	Negativ	128	113 (88.3%)		15 (11.7%)			
	Positiv	11	5 (45.5%)		6 (54.5%)			
	Extragenital localisation						0.08	0.334
	Negativ	124	104 (83.9%)		20 (16.1%)			
	Positiv	15	14 (93.3%)		1 (6.7%)			
**Mycoplama genitalium**	Genital and extragenital localisation						0.01	0.875
	Negativ	127	108 (85.0%)		19 (15.0%)			
	Positiv	12	10 (83.3%)		2 (16.7%)			
	Genital localisation						0.05	0.538
	Negativ	130	111 (85.4%)		19 (14.6%)			
	Positiv	9	7 (77.8%)		2 (22.2%)			
	Extragenital localisation						0.06	0.460
	Negativ	136	115 (84.6%)		21 (15.4%)			
	Positiv	3	3 (100.0%)		0 (0.0%)			

*V*–V Cramer size effect, *p*–*p*-value.

## Data Availability

The data that support the findings of this study are available from the corresponding author upon reasonable request.
